# Clinical Conversations on Diseases of the Skin*Cases shown and remarks made at the Demilt Dispensary, New York.

**Published:** 1876-04

**Authors:** L. Duncan Bulkley

**Affiliations:** New York


BUFFALO

### Medical and Surgical Journal.

VOL XV. APRIL, 1876. No. 9.

### Original Communications.

:o:

ART. I.—*Clinical Conversations on Diseases of the Skin.** By L. DUNCAN BULKLEY, A. M., M. D., of New York.

Reported by Robert Campbell, M. D., Clinical Assistant.

The first case, gentlemen, to which I wish to call your attention is one which, although you will not often be called upon to treat it, is important from two points of view, first, from its comparatively harmless nature as compared with syphilis, for which it might easily be mistaken, and second, in regard to its prophylaxis because of its tendency to recur.

CASE I.—ERYTHEMA PAPULATUM.—Ellen Murphy, an apparently healthy servant girl of 22 years, has twice previously had attacks of the same disease here exhibited, at intervals of nearly or about one year, that is, two years and one year ago respectively ; in the intervals she has enjoyed good health. The present attack commenced four days ago, the back of the hands and wrists being first affected and the eruption appearing on the dorsum of the feet on the following day. You now see on the backs of the hands and extending up on to the arms an eruption consisting of isolated and distinct spots of a rather pinkish violet color, varying in size from that of a small pin-head to half an inch in diameter,

*Cases shown and remarks made at the Demilt Dispensary, New York.

mostly circular, slightly elevated both to the sight and touch, and ending abruptly in healthy skin, that is, there is no shading off of the disease into healthy tissue. When I press upon these individual efflorescences they are found to pale entirely, but on suddenly removing the finger the color quickly returns, and, moreover, you can appreciate by the touch that the spots do not entirely disappear under the pressure, but that there is some element there which offers slight resistance. The surface of these blotches are smooth and slightly shining, with no trace of any desquamation. Both hands and both fore-arms present much the same appearance, the eruption being much more scattered on the latter, and the feet are similarly affected, but she tells us that the disease exists nowhere else except on the face, where it is plainly seen. Here the eruption presents somewhat different features ; on the right side of the face, especially below the eyes, are several patches of erythema of different sizes and shapes, and here, below the ramus of the jaw are two patches which are longer than broad, and none of them are as red as those on the hands, nor so clearly defined ; but pressure causes the redness entirely to disappear and they are but another phase of the same disease, which here, from the raised borders might be termed *erythema marginatum.* The entire eruption causes her but little annoyance save the unsightly appearance, for you know that almost every one is very sensitive about any spot or blemish on the skin, far more so, it is sad to say, than the same persons often are about the like affecting their character.

In regard to the family history of this patient, she tell us that there have been no eruptions in the family, (it is difficult to get this class of patients to acknowledge that there ever is), her father had lumbago, her mother sciatica and was asthmatic, but there is no history of rheumatism in the family, nor in this patient. Her general health is good, the bowels are constipated.

Those of you unacquainted with the more recent views may be surprised at my associating the term *papulatum* with *erythema,* inasmuch as a leading feature of erythematous eruptions or blushes, as they are often spoken of, is simply the *congestion,* as distinguished from eruptions attended with *exudation* on the surface or with thickening of the skin. But Hebra has ranked several species of eruptions together under the name *erythema exudativum multiforme*, and one of these in this papular form here exhibited, which he has very clearly described. This writer makes other forms of erythema to be but developments of this, which may go on even to the production of vesicles, and you will doubtless have an opportunity ere long of seeing here some cases where the congestive process has proceeded even to the effusion of so much fluid that the epidermis has become raised in blisters or small bullæ.

As I remarked at first, it is well to know and recognize these simple eruptions of the skin perfectly, for it would be a serious error to attribute this lesion to syphilis, whose large papular form this on the hands and arms resembles not a little. The differential diognostic marks are, (besides of course the absence of history of the disease), the lighter color of the eruption ; its involvement only of the hands and feet, whereas a syphilitic eruption of this intensity would certainly affect the body as well ; the more plainly erythematous character of the eruption on the face, which could never be mistaken for a syphilide ; and finally, the recurrence of the eruption at stated intervals and seasons of the year, in which this class of affections is known to appear—I know of no other eruption with which it could be confounded.

The last points to be considered are the simplest and yet the most important, namely the prognosis and therapeutics of the disease. It is self-limited and if left to itself will certainly disappear, according to Hebra, in from one to four weeks; it cannot in any way endanger life or health. Its duration, however, may be shortened, I believe, very considerably by treatment, and that which I usually advise is the magnesia and iron mixture which you hear me order as Startin's, although I am not sure that the formula is just like his. She will receive, ℞ Magnesiæ Sulphatis ℥i, Ferri Sulph. ʒi, Acid. Sulph. arom. ℥ss, Tinct. Gent. ℥i, Aduæ font. ad ℥iv.; M. Sig. Teaspoonful after meals, in water. Locally nothing is required, though in private practice it would be well to order a cooling lotion such as the following: ℞ Pulv. Calaminæ prep. ʒi, Zinci oxidi ʒi, Glycerini ʒii, Aquæ Flor. Aurant ℥iv.; M. which will have the effect also of covering up the blotches by the thin coating of flesh-colored powder which it leaves on the surface; it is of course to be well shaken before being applied.

In regard to prophylaxis against future attacks I have not much to advise, though I think that if next autumn she were to guard the health assiduously, paying particular attention to keeping the bowels relaxed and to use continuously some of the alkaline waters, together with keeping the skin in an active, healthy condition by baths, perhaps alkaline, with friction, she would be very likely to escape another attack; otherwise she may expect one.

The next case is one which it will be well to examine closely, because it demonstrates clearly the clinical differences beeween lichenoid eczema and the tinea circinata or ring worm of the body, which latter I thought this eruption to be when we first exposed a portion of the arm.

CASE II. *Eczema lichenoides resembling tinea circinata.*--This little boy, three years and a half old, comes to us for an eruption over the deltoid muscle of the left arm, extending from near the shoulder for a distance of about two inches down the arm. It is quite regularly oval in shape and of a dusky red color, composed of papules as you see on close examination, with the tops scratched off, so that the surface is rough, and, on pinching up the skin it is found to be considerably thickened. Inquiring, as you always hear me do, if this is all the eruption and if the rest of the skin is perfectly smooth, we learned that there was some eruption on the hips, and on inspection we find typical patches of dry, papular eczema. Here on either hip and over the upper part of the sacrum are spots of a yellowish hue, margins irregularly defined and with numerous distinctly papular elements.

The following are some of the reasons to be applied to diagnose the eruption before us from the tinea circinata, the herpes circinatus of older writers, which is entirely local in origin and due to the growth and development of the vegetable parasite *trichophyton* in the epithelium and hairs of the part. First, the border of the eruption is irregular in shape and not well defined, fading here in some places gradually into a healthy skin, while the margins of the parasitic affections are sharply cut, being due to the actual presence of the foreign element, the skin being abrubtly healthy beyond the place of its encroachment; as to the irregular border in the present patch we know that the vegetable parasitic diseases spread centrifugally and present a more even, circular margin, not broken into in places, as this is ; second, the eruption here is very itchy, as the scratched papules show; third, there are no scales, but on the contrary a roughness due to distinct papulation, whereas the surface within the margins of a spot of ring-worm present a uniform appearance, smooth, with a certain amount of rather easily detatched scales; fourth, the existence of other portions of disease on another part of the body, namely the hips, presenting characters very plainly distinctive of eczema; fifth and lastly, if you should scrape the surface and examine the debris from this patch under the microscope, you would fail to find the spores and mycelium composing the parasite found in tinea circinata. Our time here does not usually permit of this examination and I deem the other features quite sufficient to establish beyond doubt, in this case, that the eruption on the arm is simply a papular eczema and not the tinea circinata which it so much resembles at first sight. Let me however, strongly urge you to use the microscope in deciding these cases in private practice, for the certainty of diagnosis thereby attained fully repays the trouble, and the determination of the parasitic or non-parasitic nature of an eruption is not a very difficult one, as I shall have occasion to demonstrate to you on another occasion, and furthermore the matter is made pretty clear in the text books.

Our little patient is a rather delicate child whose nutrition is evidently below par. I will order him cod-liver oil internally, which is one of our very best remedies in many of these diseases of the skin; locally he will receive the following ointment, with directions to have it firmly rubbed into the parts night and morning: ℞ Ung. citrin. ʒiss. Ung. simpl. ʒvi. M; in private practice this should be perfumed, as with a drop or two of oil of bergamot or bitter almonds. Remember that this ointment is directed for a hard, dry, chronic patch of papular eczema, which has never given a history of having been moist, such a prescription used on an acute eczema might produce an untold amount of irritation.

This next little patient exhibits just such an eruption, which injudicious treatment might kindle up into a disease distressing beyond measure to child, family and doctor, but which is doing very well under the very different measures which have been advised at former visits.

CASE III. *Impetiginous eczema of cheeks and lower extremities.*—Albert N. aged 2½ years, has had an eruption similar to the present one since he was six months old, it dissappearing at times, to reappear soon again. The father of the child has asthma, the mother has been subject to furuncles, as also the eldest brother, now five years of age, and the child's maternal grandfather has had rheumatism. This little one therefore, comes naturally by its eczema and also its asthmatic breathing, especially after eating, a not very uncommon affection in children with this form of eczema.

At present there is an impetiginous eruption covering the patients cheeks, with a tendency to the formation of crusts. The skin here presents the characteristic appearance of a moist, reddened surface, very itchy, with more or less of a pustular element here and there. On the back you find the skin far from normal, it being very papular, hard and dry, and evidently gives the patient great annoyance from the itching. The inner and posterior surface surfaces of both legs are covered with a dry and scaly eruption, quite punctate, with here and there groups or patches of disease composed of aggregated papules. The eruption is on the decline, and you have heard the mother say that she believes that the internal remedies have effected a great change, and that she has not been very faithful in the use of the local treatment. The child from the first was put upon DeValangin's solution of arsenic (liquor arsenici chloridi), it being prescribed diluted with equal parts of cinnamon water; commencing with four drops of the mixture after meals, the dose has been increased by a drop a day until now he takes ten drops of the arsenic, or 20 of the mixture, three times a day, further diluted, of course, in a teaspoonful or so of water. Locally he has had only the oxide of zinc ointment; he has had no other remedies.

Now some may think that this is at variance with what I have previously said in reference to a routine treatment of eczema with arsenic and zinc ointment, but quite the contrary is the case. What I would guard you against is getting into the rut traversed by so many, who, as soon as the diagnosis of eczema is made, prescribe as it were necessarily, these remedies, whereas you will see comparatively, but very few cases, treated by me with these two agents alone, or even combined with others. There are reasons in this case for the employment of them, and I think it would be as great an error for you not to use this treatment *when it is indicated*, as to employ it when it is not required.

The reasons, then, for giving this child this line of treatment are: first, the arsenic is called for by the itchiness of the eruption, for you have had abundant confirmation from the mouths of patients and from the parents of little patients here, to show, that when under the full influence of arsenic there is less irritation of the skin, the sleep is better and the eruption consequently improves, with or in consequence of local treatment; second, the child has asthmatic breathing and the father is distressed at times with the same, and arsenic certainly does alieviate if not remove this, in proper cases, that is, in those not due to organic disease, as that of the child assuredly is not; third, the rheumatism in the family, together with the appearance of the child and its history, and that of the eruption mark the case as one of the arthritic variety of eczema, which, as the French especially have taught us, is benefitted by arsenic and alkalies. We have tried the arsenic first and alone, and the success confirms the theory; the alkalies have not been given because, as a rule, children do not require them so much as adults, there is not the tendency to hyper-acidity that we find in those of older years. The zinc ointment was given merely because it is one of the very best mildly stimulating and yet soothing applications; and, furthermore, it prevents the friends from using worse things, and, if well applied, it serves perfectly to exclude air and dust and to keep the surface from drying up and crusting. We will continue the same treatment, there are no indications for a change.

